# Generation of ozone during irradiation using medical linear accelerators: an experimental study

**DOI:** 10.1186/s13014-022-02005-6

**Published:** 2022-02-22

**Authors:** N. Hara, J. Oobuchi, A. Isobe, S. Sugimoto, J. Takatsu, K. Sasai

**Affiliations:** 1 Department of Radiology, Juntendo University Hospital, Tokyo, 113-8431 Japan; 2 Department of Radiation Oncology, Graduate School of Medicine, Juntendo University, 2-1-1, Hongo, Bunkyo-ku, Tokyo, 113-8421 Japan

**Keywords:** Ozone, Phantosmia, Medical linear accelerator, X-ray, Electron beams

## Abstract

**Background:**

Some patients have noted a foul odor during radiation therapy sessions, but the cause of the odor remains unknown. Since we suspected that this phenomenon is due to ozone generated by ionizing radiation, this experimental study measured ozone concentrations in the treatment room and in a coiled polyvinyl chloride (PVC) tube placed within the radiation field.

**Methods:**

We measured ozone concentrations using an ultraviolet absorption method and an ozone monitor. A PVC tube (inner diameter 7 mm, outer diameter 10 mm) was used to mimic the environment of the nasal cavity. The tube (790 cm) was coiled and set between two 4-cm-thick (for X-rays) or 2-cm-thick (for electron beams) water-equivalent solid phantoms. The sampling tube of the ozone monitor was inserted into the PVC tube, and the joint was sealed to prevent environmental air contamination. To measure ozone concentrations in the atmosphere, the sampling tube supplied with the unit was used. A linac was used on a full-sized treatment field (40 cm × 40 cm at a source-to-axis distance of 100 cm). The effect of an electron beam on ozone concentrations was also evaluated with a full-sized treatment field (40 cm × 40 cm at a source-to-surface distance of 100 cm).

**Results:**

Ozone levels in the treatment room were undetectable before the start of daily treatment but reached 0.008 parts per million (ppm) or more at 1 h after the start of treatment. Concentrations then remained nearly constant at 0.010–0.015 ppm throughout the day. The maximum ozone concentration in the PVC tube was only 0.006 ppm, even when it was irradiated at 2400 monitor units/min. Depending on the X-ray dose rate, the concentration increased to a maximum of 0.010 ppm with oxygen flowing into the other end of the tube at 1.5 L/min. Ozone concentrations in the PVC tube did not differ significantly between X-ray and electron-beam irradiation.

**Conclusions:**

Only traces of ozone were found in the PVC tube that was used to mimic the nasal passages during radiation, these concentrations were too low for human perception. However, ozone concentrations did reach potentially detectable levels in the treatment room.

**Supplementary Information:**

The online version contains supplementary material available at 10.1186/s13014-022-02005-6.

## Background

Some patients perceive a foul odor during radiation therapy sessions, but the cause of this phenomenon remains unclear [1–3]. Several participants in the cancer survivor network of the American Cancer Association stated that they noticed unpleasant odors during radiation sessions, with some claiming that the odor was similar to that of ozone [4]. Our previous retrospective chart review demonstrated that the incidence of this phenomenon was less than 5% [3]. However, a much higher incidence was reported in the 1990s [1]. Moreover, in a recent prospective study, we found that approximately 34% of brain tumor patients reported such an odor [5].

Two theories have been proposed, i.e., that substances such as ozone generated by radiation are responsible for the odor [1, 6] and that no odor actually exists (phantosmia) [2]. We recorded the times at which patients reported an odor during a treatment session with a helical TomoTherapy apparatus, and we found that almost all reports occurred in instances when the treatment beam passed through the olfactory epithelium and/or ethmoid sinuses [5]. Although this implies that a substance such as ozone, generated by X-radiation of these areas, was detected by the epithelium, the possibility remains that the phenomenon is caused by stimulation of the nervous system by ionizing radiation.

Costello et al. reported a maximum ozone concentration of less than 0.015 parts per million (ppm) in a treatment room [7]. Furthermore, the compound was undetectable under typical radiation treatment conditions.

Based on the results of our observational study [5], we suspected that the pungent smell experienced during the radiation session is caused by ozone generated by the X-ray itself or its secondary electrons. Thus, we designed an experimental study in which we measured ozone concentrations in the treatment room and in the field of radiation.

## Materials and methods

Ozone concentrations were measured by an ozone monitor (Model 1150; Dylec Inc., Ibaraki, Japan) using an ultraviolet absorption method [8, 9]. The sample air inflow rate of this device is 1.5 L/min. The minimum detectable concentration of ozone is 0.001 ppm, and measurements are accurate to within 0.001 ppm. To measure ozone concentrations in the atmosphere, the sampling tube supplied with the unit was used. To measure the ozone concentrations in the polyvinyl chloride (PVC) tube described below, the sampling tube was inserted into the PVC tube, and the joint was sealed to prevent environmental air contamination. To measure ozone levels in the treatment room, the device was placed at a height of 80 cm from the floor, 3 m from the beam isocenter at 210 degrees (where 180 degrees corresponds to the caudal direction).

**Fig. 1:**
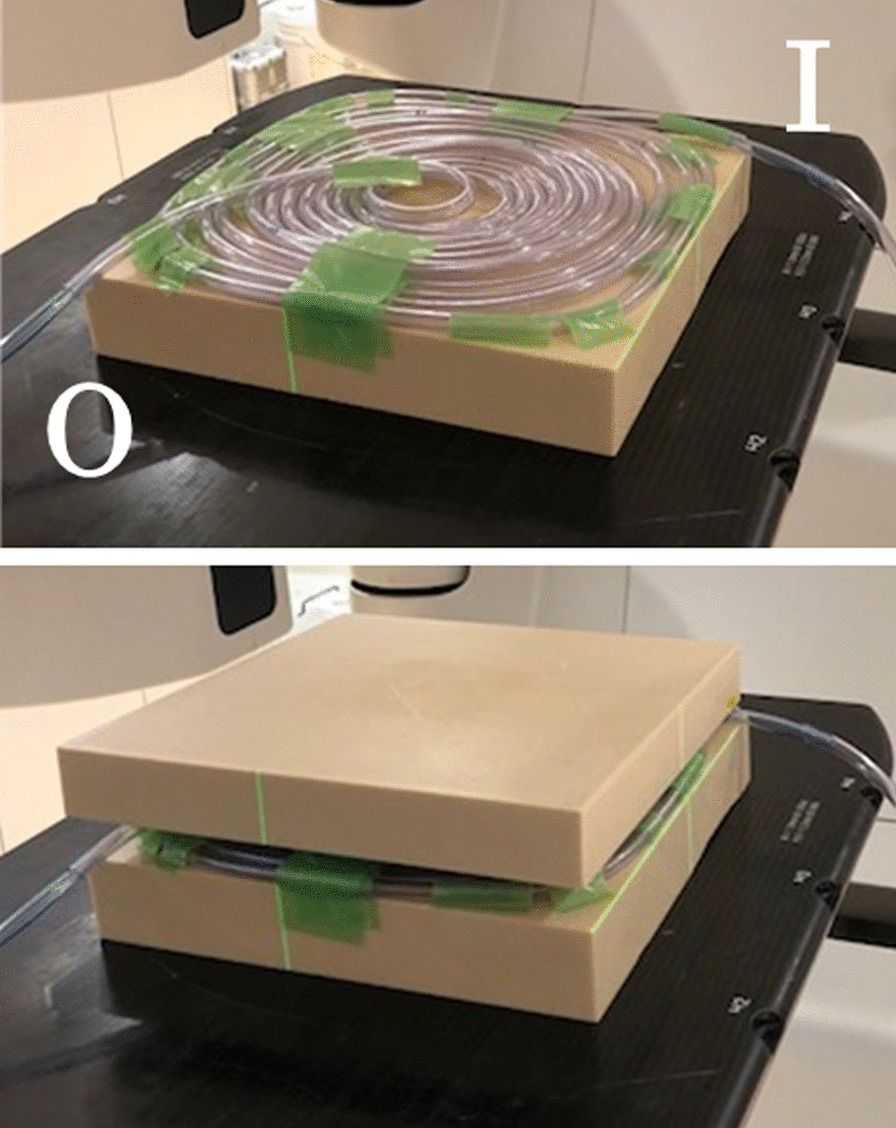
A coil of polyvinyl chloride tube (outer diameter: 10 mm; inner diameter: 7 mm, length 790 cm) was placed between two 4-cm-thick water-equivalent solid phantoms. O: outlet to the ozone-measuring device; I: inlet for air and oxygen

A PVC tube (inner diameter 7 mm, outer diameter 10 mm; Makiguchi Rubber, Hongo, Japan) was used to mimic the environment of the nasal cavity. The tube (790 cm) was coiled and set between two 4-cm-thick (for X-rays) or 2-cm-thick (for electron beams) water-equivalent solid phantoms, as shown in Fig. 1.

Two medical linear accelerators (linacs) (TrueBeam: Varian, Palo Alto, CA, USA; TomoTherapy HD: Accuray, Sunnyvale, CA, USA) were used to measure ozone concentrations in the treatment field. The TrueBeam was used with a full-sized treatment field (40 cm × 40 cm at a source axis distance of 100 cm). To evaluate the dose-rate dependence of ozone concentrations, the phantom was irradiated with X-rays at 400, 600, 1400 and 2400 monitor unit (MU)/min [6 MV flattening filter (FF), 10 MV FF, 6 MV FF free (FFF) and 10 MV FFF)]. The effect of an electron beam on ozone concentrations was also evaluated with a full-sized treatment field (40 cm × 40 cm at a source surface distance of 100 cm). The phantom was irradiated with 6-, 9-, 12-, and 15-MeV electron beams at dose rates of 400–2500 MU/min.

TomoTherapy was applied using a jaw field of 25.1 mm and a pitch of 0.43, mimicking the treatment conditions of patients who complained of a foul odor.

Daily variations in ozone concentrations during treatment were also measured in treatment rooms equipped with TrueBeam and TomoTherapy machines. Excluding the maze entrances, the two treatment rooms measure 6.7 m × 7.25 m × 3.05 m and 6.7 m × 7.35 m × 3.35 m in size. The ventilator capacity in each room was 300 m^3^/h. The air in the room was replaced approximately three times per hour.

On the 2 workdays when ozone levels were measured in the treatment rooms, the TrueBeam machine emitted 16,000 MU on one day and 15,700 MU on the other, and the TomoTherapy machine emitted 65,400 MU on one day and 49,700 MU on the other.

## Results

**Fig. 2:**
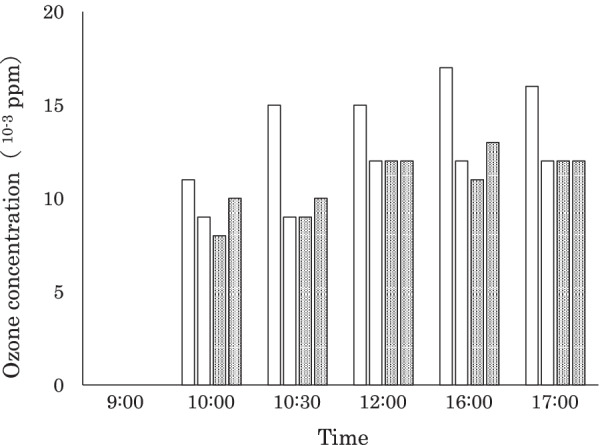
Mean ozone concentrations in the treatment rooms. White bars denote the concentrations in the room equipped with the TrueBeam machine, and dotted bars denote the concentrations in the room equipped with the TomoTherapy machine. Concentrations were measured on 2 different days. The standard deviations of the three measurements are too small to display

Figure 2 shows the changes in ozone concentrations in the two treatment rooms equipped with the TrueBeam and TomoTherapy machines. There was no detectable ozone in the morning before treatment began. However, in both rooms, concentrations exceeded 0.008 ppm at 1 h after the start of treatment and ranged from 0.010 to 0.015 ppm throughout the day.

**Fig. 3:**
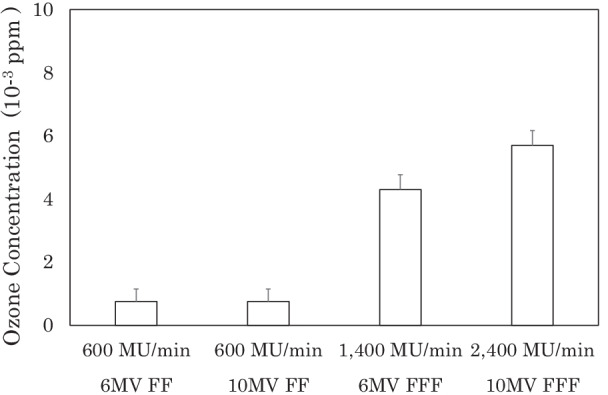
Mean ozone concentrations in the PVC tube according to the beam energy and dose rates of X-rays from a medical linac (TrueBeam). The error bars denote the standard deviations of the three measurements. FF: flattening filter; FFF: flattening filter free; MU: monitor unit

**Fig. 4:**
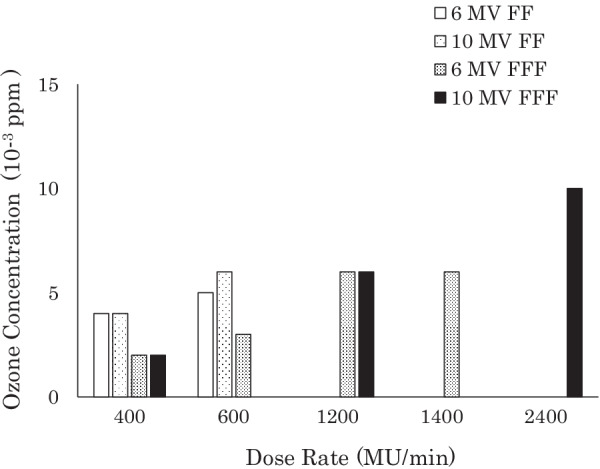
Mean ozone concentrations in the PVC tube according to the beam energy and dose rates of X-rays from a medical linac (TrueBeam) while oxygen flowed into the end of the tube at a rate of 1.5 L/min. The standard deviations of the three measurements are too small to display. FF: flattening filter; FFF: flattening filter free; MU: monitor unit

**Fig. 5:**
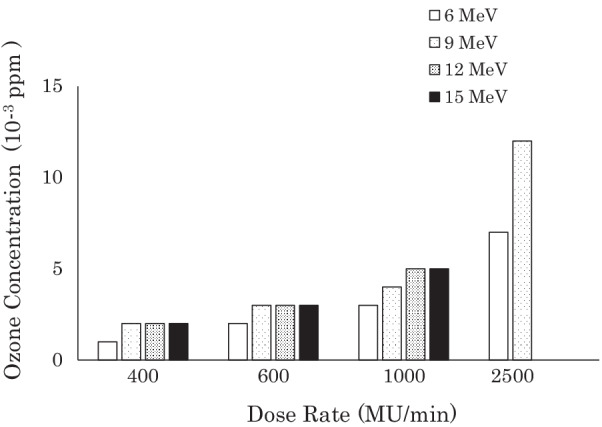
Mean ozone concentrations in the PVC tube according to the beam energy and dose rates of electron beams from a medical linac (TrueBeam) while oxygen flowed into the end of the tube at a rate of 1.5 L/min. The standard deviations of the three measurements are too small to display. MU: monitor unit

Figure 3 shows the ozone concentrations in the PVC tube according to the radiation dose rate and X-ray energy. The maximum concentration was only 0.006 ppm, even when the tube was irradiated at 2400 MU/min. The same experiment was then performed with 1.5 L/min of oxygen flowing into the other end of the tube under the assumption that the patient would occasionally inhale oxygen during radiation therapy. This flow rate was the same as the inflow rate of the ozone-measuring unit. The ozone concentration increased by up to 0.010 ppm depending on the X-ray dose rate (Fig. 4). Next, concentrations were measured when the tube was irradiated with the electron beams. Figure 5 shows the results: ozone concentrations were nearly the same as during irradiation by X-rays.

We also measured the ozone concentrations in the tube when set in the same positions as the nasal passages of three randomly selected patients who were treated with TomoTherapy and complained of foul odors [5]. However, no trace of ozone was detected in any case.

## Discussion

In this study, we measured the ozone concentration in a PVC tube, which is far different from the human nasal cavity. We used an ozone monitor that needs a sample air inflow rate of 1.5 L/min. This makes it difficult to measure the ozone concentration in a small cavity mimicking the nose. Therefore, we used a long tube, which could supply a sufficient amount of irradiated sample to the device.

Ozone can be detected by humans at concentrations above 0.015–0.020 ppm [10]. Although ozone was generated by X-rays in this study, the concentrations in the PVC tube were too low for human perception, implying that the source of the possible odor is elsewhere. However, we measured concentrations only in air; thus, it is possible that ozone generated in the mucosa stimulates olfactory receptors differently, as suggested by Sagar et al. [1]. They noted the chemical reactions between ozone and other radicals in the mucosa close to the olfactory receptors; reaction products such as hydroxy radicals can stimulate the membrane where the receptors are located.

Ionizing radiation can synthesize ozone in air [11–13]. However, a search of PubMed on April 15, 2021, using the search terms “ozone” AND (“linac” OR “linear accelerator” OR “electron beam” OR “radiotherapy” OR “radiation therapy”) revealed only two studies on the generation of ozone by medical linacs. Holloway and Cormack calculated ozone concentrations during treatment with a linac and concluded that concentrations remained low, except in exceptional circumstances [14].

Costello et al. reported measurements of ozone obtained in a treatment room and in a direct radiation beam [7]. Although they did not provide details of the study setup in their short report, their results mirror ours. They found that the maximum concentration in the treatment room was less than 0.15 ppm. However, this concentration is higher than the safe level of this compound, as described below; therefore, we suspect that the reported value may have been a typographical error. They did not detect ozone during direct widefield irradiation with photons and electrons. They measured the concentration of ozone in a structure mimicking the nasal passages, but no ozone was detected there.

The maximum ozone concentration in either treatment room was 0.015 ppm in our study, which was significantly higher than the concentration in the treatment field. In a previous study, a young patient complained of a foul odor not only during radiation sessions but also upon approaching the corridor leading into the radiotherapy room [3]. It was suggested that the perceived odor could have been due to a learned response akin to anticipatory vomiting in patients who receive chemotherapy. However, our study clearly demonstrated that treatment-room ozone concentrations can reach levels detectable by humans. Although staff did not detect the odor, the young patient may have been better able to perceive it due to the superior sense of smell of juveniles compared to adults [15].

In this study, the concentrations of ozone in the treatment rooms were not detectable before the use of the linacs but significantly increased after treatment began (Fig. 2). Concentrations were much higher in the room than in the direct treatment beam. In the PVC tube mimicking the nasal cavity, X-rays and electrons drive the synthesis of ozone from only a small amount of air or oxygen, whereas in the treatment room, the volume of irradiated air is much larger. This may have contributed to the difference in ozone concentrations seen in this study. Regardless, ozone concentrations in the treatment rooms were far lower than the upper safety limit of 0.1 ppm [16].

An old guideline states that ozone production during X-ray therapy is negligible. However, electron beams from radiotherapy accelerators produce ozone [11]. In our study, X-rays and electron beams synthesized ozone in almost identical amounts. We measured the ozone concentration in a PVC tube after allowing the radiation to build up to full intensity (Fig. 1); therefore, it is thought that the electron density inside the tube was stable.

The ozone concentrations in the tube were lower upon irradiation with FFF beams than with FF beams (Fig. 4). FF beams deliver homogeneous dose distributions, whereas FFF beams deliver the peak dose to the central axis; doses decrease as distance from the axis increases, especially in a large field [17]. We confirmed this phenomenon under our experimental conditions using a computer simulation (Additional file 1: Figures). We irradiated the PVC tubes in a 40 cm × 40 cm field; therefore, some volume of air was irradiated below the peak dose rate when FFF beams were used. A dosimetric analysis demonstrated that the mean absorbed dose by the sample air in the tube was approximately half as large with FFF beams as with FF beams (6 MV X-rays: FFF 62.5 cGy, FF 102.1 cGy; 10 MV X-rays: FFF 49.8 cGy, FF 104.6 cGy upon irradiation with 100 MU). The difference in the ozone concentration between the FF beams and FFF beams reflected this phenomenon. Our dose simulation also demonstrated that the absorbed doses were almost exactly the same between 6 and 10 MV X-rays (Additional file 1: Figures); therefore, there was no difference in the amounts of generated ozone between the two X-ray energy levels.

Gerasimov reported that the amount of ozone generated by ionizing radiation depends on the concentration of oxygen in the air [12]. Although the concentration of ozone generated by X-rays in normal air does not reach the human-detectable limit, we were interested in whether it could reach that level in the case of supplemental oxygen administration, as patients sometimes need supplemental oxygen during radiation sessions due to their medical conditions. However, the ozone concentration increased by up to 0.010 ppm (Fig. 4), which was still lower than the human-detectable level.

## Conclusions

We found that the maximum ozone concentration in the PVC tube was 0.006 ppm, which is too low to be perceived by humans. However, the maximum in the treatment room was 0.015 ppm, which humans can perceive.

## Supplementary Information

**Additional file 1.** The dose profiles at a depth of 4 cm in water were compared among 6 MV FF, 6 MV FFF (Fig. 1), 10 MV FF, and 10 MV FFF (Fig. 2). Dose profiles were calculated using a radiation treatment planning system (Eclipse ver. 13.6, Varian, Palo Alto CA, USA) with a dose grid size of 2 mm. The plan parameters were as follows: source-to-surface distance (SSD), 96 cm; field size, 40 x 40 cm^2^. The same monitor unit (MU) was irradiated (100 MU). The analytical anisotropic algorithm (AAA) was used as the dose calculation algorithm.

## Data Availability

The datasets used and/or analyzed during the current study are available from the corresponding author.

## Abbreviations

FF: Flattening filter
FFF: Flattening filter free
linac: Linear accelerator
MU: Monitor unit
PPM: Parts per million
PVC: Polyvinyl chloride
