# Supplementary material for: Generation of ozone during irradiation using medical linear accelerators: an experimental study
Source: Radiat Oncol. 2022 Feb 22;17:39. doi: 10.1186/s13014-022-02005-6 (PMC8864795; doi:10.1186/s13014-022-02005-6)
Supplement: Supplementary file 1 — Additional file 1. The dose profiles at a depth of 4 cm in water were compared among 6 MV FF, 6 MV FFF (Fig. 1), 10 MV FF, and 10 MV FFF (Fig. 2). Dose profiles were calculated using a radiation treatment planning system (Eclipse ver. 13.6, Varian, Palo Alto CA, USA) with a dose grid size of 2 mm. The plan parameters were as follows: source-to-surface distance (SSD), 96 cm; field size, 40 x 40 cm2. The same monitor unit (MU) was irradiated (100 MU). The analytical anisotropic algorithm (AAA) was used as the dose calculation algorithm. [file 13014_2022_2005_MOESM1_ESM.pdf]

Additional file 1: Figure 1

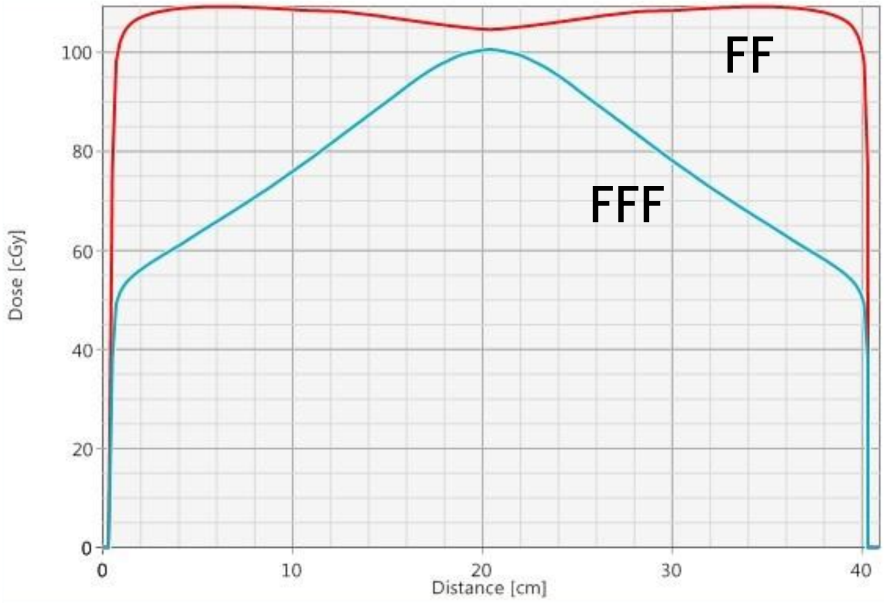

Additional file 1: Figure 2

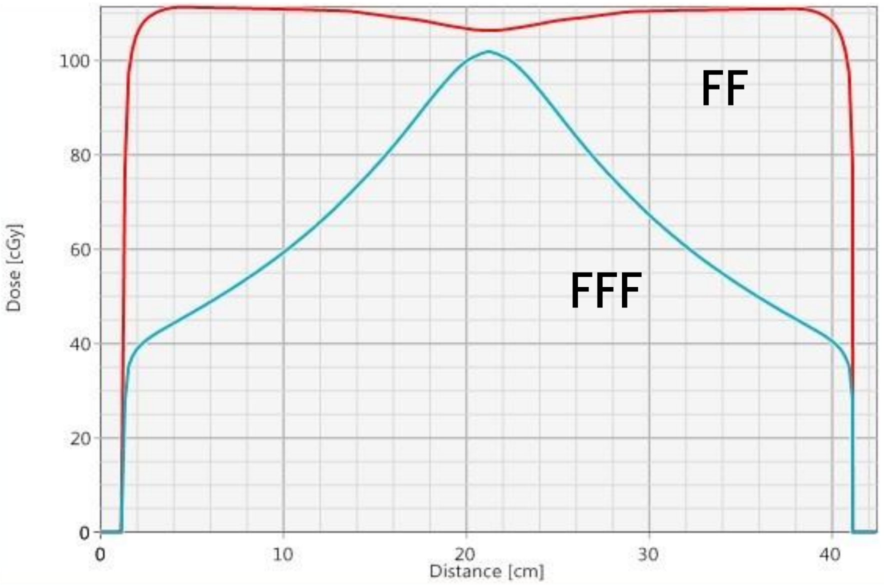

The dose profiles at a depth of 4 cm in water were compared among 6 MV FF, 6 MV FFF (Fig. 1), 10 MV FF, and 10 MV FFF (Fig. 2). Dose profiles were calculated using a radiation treatment planning system (Eclipse ver. 13.6, Varian, Palo Alto CA, USA) with a dose grid size of 2 mm. The plan parameters were as follows: source-to-surface distance (SSD), 96 cm; field size, 40 x 40 cm^2. The same monitor unit (MU) was irradiated (100 MU). The analytical anisotropic algorithm (AAA) was used as the dose calculation algorithm.
